# A rare variant of the ulnar artery with important clinical implications: a case report

**DOI:** 10.1186/1756-0500-5-660

**Published:** 2012-11-30

**Authors:** Diogo Casal, Diogo Pais, Tiago Toscano, Tiago Bilhim, Luís Rodrigues, Inês Figueiredo, Sónia Aradio, Maria Angélica-Almeida, João Goyri-O’Neill

**Affiliations:** 1 Department of Anatomy, Faculty of Medical Sciences, New University of Lisbon, Lisbon, Portugal; 2 Plastic and Reconstructive Surgery, São José Hospital, Lisbon 1150-199, Portugal; 3 Department of Anatomy, Faculty of Medical Sciences, New University of Lisbon, Campo dos Mártires da Pátria, 30, 1169-056, Lisbon, Portugal; 4 Plastic and Reconstructive Surgery, Santa Maria Hospital, Avenida Professor Egas Moniz, 1649-035, Lisbon, Portugal; 5 Faculty of Medical Sciences, New University of Lisbon, Campo dos Mártires da Pátria, 30, 1169-056, Lisbon, Portugal; 6 Plastic and Reconstructive Surgery Department and Burn Unit, São José Hospital, Lisbon, 1150-199, Portugal

**Keywords:** Blood supply, Anatomy, Surgery, Arteries, Arm, Forearm, Cadaver, Dissection

## Abstract

**Background:**

Variations in the major arteries of the upper limb are estimated to be present in up to one fifth of people, and may have significant clinical implications.

**Case presentation:**

During routine cadaveric dissection of a 69-year-old fresh female cadaver, a superficial brachioulnar artery with an aberrant path was found bilaterally. The superficial brachioulnar artery originated at midarm level from the brachial artery, pierced the brachial fascia immediately proximal to the elbow, crossed superficial to the muscles that originated from the medial epicondyle, and ran over the pronator teres muscle in a doubling of the antebrachial fascia. It then dipped into the forearm fascia, in the gap between the flexor carpi radialis and the palmaris longus. Subsequently, it ran deep to the palmaris longus muscle belly, and superficially to the flexor digitorum superficialis muscle, reaching the gap between the latter and the flexor carpi ulnaris muscle, where it assumed is usual position lateral to the ulnar nerve.

**Conclusion:**

As far as the authors could determine, this variant of the superficial brachioulnar artery has only been described twice before in the literature. The existence of such a variant is of particular clinical significance, as these arteries are more susceptible to trauma, and can be easily confused with superficial veins during medical and surgical procedures, potentially leading to iatrogenic distal limb ischemia.

## Background

A sound knowledge of the vascular anatomy of the upper limb is of paramount importance, since this is a site of frequent injury and of various surgical and invasive procedures [1,2]. Normally, the arterial supply to the upper limb is provided by the axillary artery that originates the brachial artery, which, in turn, at the elbow originates the ulnar and radial arteries [3]. These two are placed between the forearm muscles, and give rise at the wrist level to the arteries that form the superficial and deep arterial palmar arches [3]. Usually, the ulnar artery gives off the common interosseous artery that divides into the anterior and posterior interosseous arteries [3].

It has been increasingly recognized that variations in the major arteries of the upper limb are common, being found in up to one fifth of individuals [1,4,5]. Among these, variants of the ulnar and radial arteries are the most common [1,3,4]. Particularly, the presence of superficial radial or ulnar arteries is of utmost clinical significance, as these arteries are most susceptible to trauma, and can be easily confused with superficial veins [1,2]. One variant of superficial ulnar arteries is the superficial brachioulnar artery (SuBUA), which is defined as an ulnar artery with a high origin in the arm that progresses over the superficial muscles of the forearm. The prevalence of the SuBUA varies widely in different studies [3]. For example, Adachi, in 1928, in an impressive series of 1198 upper limb dissections, identified only 8 cases of SuBUA, corresponding to a 0,7% prevalence of this variant [6]. In contrast, in 1844, Quain, had found 7% of SuBUA in 429 specimens dissected [7]. According to a recent review by Rodriguez-Niedenfuhr et al., the overall prevalence of this variant in the literature is estimated to be around 2,7% [3].

The authors report the case of a cadaver in which a bilateral SuBUA with an unusual path was identified bilaterally. The clinical implications of this anatomical variation are undoubtedly of great significance [3,5,8], and are described briefly in the Discussion Section.

## Case presentation

During routine dissection of a 69-year-old fresh female cadaver at the Department of Anatomy at our institution, variations in the arterial system of both upper limbs were noted. There was no history or evidence of any invasive procedure in the upper limbs of that person.

**Figure 1:**
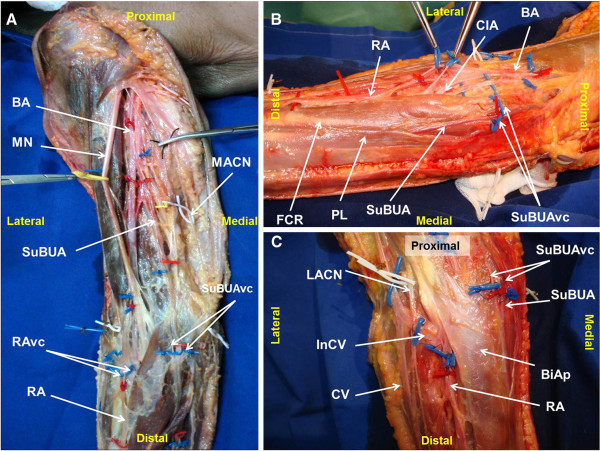
**Right upper limb dissection photographs showing the origin and path of the superficial brachioulnar artery, and their neighbor structures at the arm and elbow. A **– Upper arm and proximal forearm; **B **– Medial aspect of the elbow region; **C**– Anterior aspect of the elbow region. RA radial artery; SuBUA superficial brachioulnar artery; BA brachial artery; CIA common interosseous artery; CV cephalic vein; InCV Intermediate cephalic vein; RAvc radial artery venae comitantes; SuBUAvc superficial brachioulnar artery venae comitantes; LACN lateral antebrachial cutaneous nerve; MACN medial antebrachial cutaneous nerve; MN median nerve; PL palmaris longus muscle; FCR flexor carpi radialis muscle; BiAp bicipital aponeurosis.

**Figure 2:**
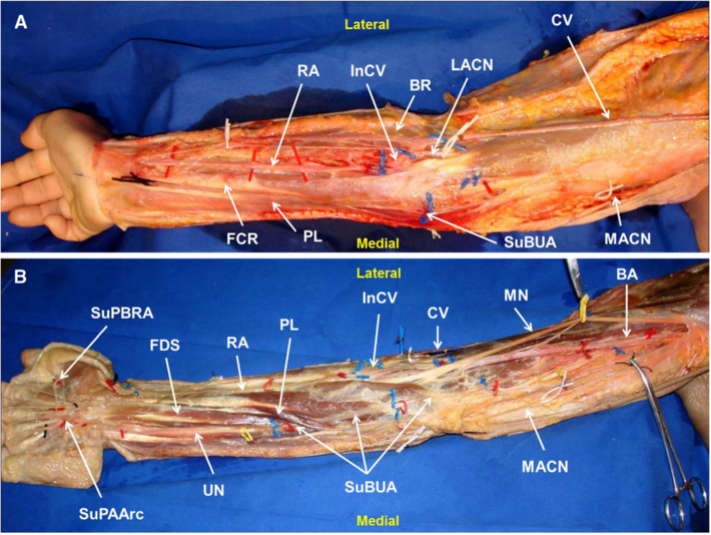
**Right upper limb showing the anomalous course of the superficial brachioulnar artery in an above fascia (A) and a deeper to fascia (B) dissection. **RA radial artery; SuBUA superficial brachioulnar artery; BA brachial artery; CIA common interosseous artery; CV cephalic vein; InCV Intermediate cephalic vein; RAvc radial artery venae comitantes; LACN lateral antebrachial cutaneous nerve; MACN medial antebrachial cutaneous nerve; MN median nerve; PL palmaris longus muscle; FCR flexor carpi radialis muscle; FDS flexor digitorum superficialis muscle.

On both sides, the brachial artery in the middle third of the arm originated a SuBUA (Figures 1 and 2). This artery penetrated the brachial fascia in the lower third of the arm, crossed anteriorly to the bicipital aponeurosis and to the muscles that originated from the medial epicondyle, and ran over the pronator teres muscle in a doubling of the antebrachial fascia (Figure 2). In the elbow region, the SuBUA was in intimate contact with the superficial structures, namely the medial antebrachial nerve and the subcutaneous veins (Figure 1). It then dipped into the forearm fascia, passed through a gap between the palmaris longus and the flexor carpi radialis, ran deep to the palmaris longus muscle belly, and superficially to the flexor digitorum superficialis muscle, reaching the gap between the latter and flexor carpi ulnaris muscle (Figure 2). In the middle third of the forearm the SuBUA was positioned lateral to the ulnar nerve.

The brachial artery continued through the radial artery (RA), which followed its usual course. In the upper third of the forearm, the RA gave off the common interosseous artery. This latter artery branched into the anterior and the posterior interosseous arteries (Figure 1). The anterior interosseous artery had a large caliber and originated branches to most of the anterior compartment muscles. The radial recurrent artery emanated from the radial artery, and the anterior ulnar recurrent artery was a branch of the common interosseous trunk.

**Figure 3:**
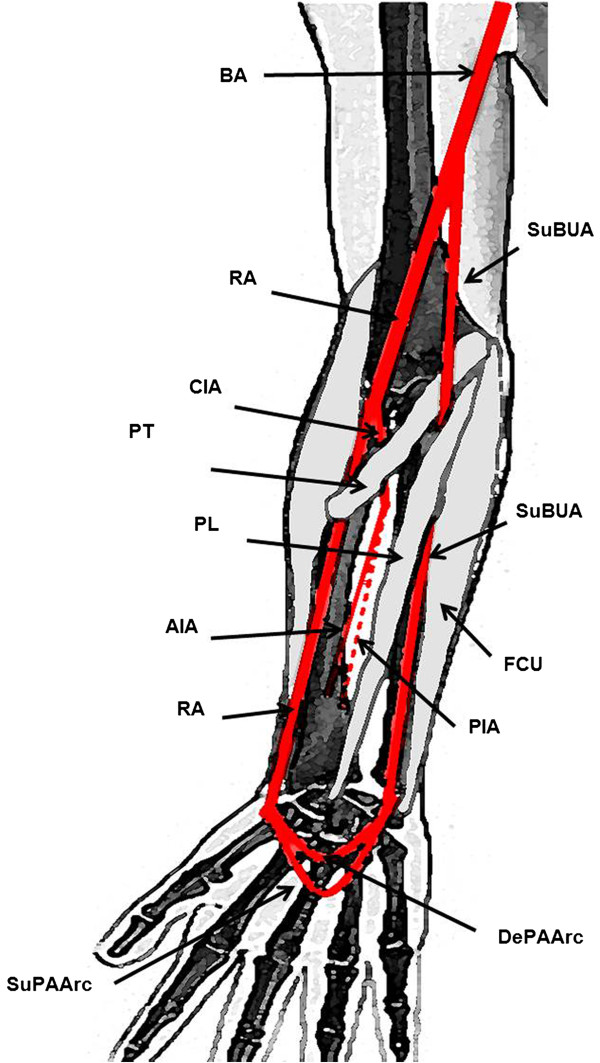
**Schematic drawing of the origin and distribution of the superficial brachioulnar artery and its relation with the medial epicondyle muscles. **RA radial artery; SuBUA superficial brachioulnar artery; BA brachial artery; CIA common interosseous artery; AIA anterior interosseous artery; PIA posterior interosseous artery; SUPAArch superficial palmar arterial arch; DePAArc deep palmar arterial arch; PL palmaris longus muscle; BR brachioradialis muscle; PT pronator teres muscle; FCU flexor carpi ulnaris muscle; PL palmaris longus muscle.

In the distal third of the forearm and in the wrist region, the RA and the SuBUA divided in the same manner as the radial and ulnar arteries usually distribute [3], originating the superficial and deep palmar arterial arches. Figure 3 schematically portrays the distribution of the RA and the SuBUA in the cadaver herein described.

## Discussion

Rodriguez-Niedenfuhr et al., have recently proposed a system of classification of upper limb arterial variations, based on their extensive experience of almost 400 upper limb dissections, and based on a thorough literature review on the subject [3,8,9]. This terminology, which recently has been taken up by several authors [5], considers each arterial variation as an individual entity along its full extension in the upper limb [3]. Furthermore, this classification divides upper limb arterial variants in three broad groups based on their location in the arm, the arm and forearm, or the forearm. These three groups are further subdivided in several different categories, depending on the absence or duplication of arteries, and on whether these variants adopt a superficial or usual course in the forearm [3].

The variations found exclusively in the forearm are the superficial brachial artery and the accessory brachial artery. The former represents a brachial artery coursing in front of the median nerve, instead of being placed behind it. The accessory brachial artery is characterized by the existence of 2 brachial arteries that rejoin before giving off the forearm arteries. The accessory brachial artery originates from the main brachial artery [3].

The variations located at the level of both the arm and forearm are the superficial brachioulnar (SuBUA), the brachioulnar, the brachioradial, the superficial brachioradial, the brachiointerosseous, the superficial brachiomedian, and the superficial brachioulnoradial arteries [3].

The SuBUA is characterized by an ulnar artery that originates higher than usual and that courses over the forearm flexor muscles. In this setting, there is a whole arterial pattern, with a brachial or superficial brachial artery branching into the radial and common interosseous arterial trunk, or more rarely into the radial and ulnar arteries [3].

The brachioulnar artery corresponds to a high origin of the ulnar artery from the brachial artery, with the latter branching into the radial artery and the common interosseous arterial trunk [3].

The brachioradial artery represents a high origin of the radial artery from the brachial or superficial brachial artery that in turn branches into the ulnar artery and the common interosseous arterial trunk [3].

The superficial brachioradial artery consists of a high origin of the radial artery coursing over the brachioradialis muscle or the tendons that limit the snuffbox. In these circumstances the brachial artery usually originates the ulnar artery and the common interosseous arterial trunk [3].

The brachiointerosseous artery is defined by a high origin of the interosseous arterial trunk, in the context of a whole arterial pattern of the upper limb, with a brachial artery that divides into the radial and ulnar arteries [3].

The superficial brachiomedian artery is characterized by a high origin of the median artery that courses above the superficial flexor muscles and by a brachial artery that divides into the radial and ulnar arteries [3].

Finally, the superficial brachioulnoradial artery represents a superficial brachial artery that at the level of the elbow branches into the radial and ulnar arteries, which in turn will course over the superficial forearm flexor muscles. In this variant, the superficial brachial artery coexists with a normal brachial artery that ends in the common interosseous arterial trunk [3].

The variations found exclusively at the forearm level include the superficial radial artery, the duplication of the radial arteries, and the absence of the radial or ulnar arteries [3].

The superficial radial artery consists of a radial artery coursing above the tendons limiting the snuffbox. The absence of either the radial or ulnar arteries is considered very rare, as is the true duplication of the radial artery [3].

Therefore, considering Rodriguez-Niedenfuhr’s classification, our case most closely resembles a SuBUA variant [3]. This variant corresponds to a brachial artery originating a superficial ulnar artery high up in the arm, whereas the radial artery is a continuation of the brachial artery [3]. The origin of the interosseous arteries from the radial artery, as recorded in the present case, is considered common in cases of ulnar arteries arising in the arm [3].

According to most authors, the SuBUA most frequently courses posteriorly to the bicipital aponeurosis, and not anteriorly as it was observed in our dissection (Figures 1C and 2A) [3]. In addition, in the work conducted by Rodriguez-Niedenfuhr et al., in all cases the SuBUA coursed anteriorly to all the flexor muscles of the forearm, and then placed itself in the lateral border of the flexor carpi ulnaris to adopt its position in the lateral aspect of the ulnar nerve at the level of the middle third of the forearm [3]. As far as the authors could determine, a SuBUA variant similar to the one we observed, with a path deep to the palmaris longus muscle, has just been reported twice in the literature. Quain found it in 2 cases while dissecting 429 upper limbs [7], and Hazlet once in 188 limbs [10].

Upper limb vascular variations are presently thought to result from a stochastic process of persistence, enlargement and differentiation of parts of the initial capillary network which would normally remain as capillaries or even regress [5,11]. The precise mechanisms that lead to the higher frequency of certain variants over others, remain to be elucidated [5,11]. Interestingly, Rodriguez-Niedenfuhr et al., identified a SuBUA in 4,7% of 150 upper limbs of embryos, which is a value superior to that reported by most authors in the general adult population [11].

The clinical importance of the superficial variations of the arteries of the upper limb are increasingly being recognized [1]. For example, by being superficial, they can be easily mistaken for subcutaneous veins, leading to inadvertent artery cannulation, with the potential risk of distal limb ischemia [1,12,13]. In addition, the superficial position of the radial or ulnar arteries makes them more vulnerable to trauma [1]. Moreover, the possibility of a SBUR variant should always be born in mind when using the arm or forearm as a source or as a recipient of microvascular flaps, or when using the radial artery as vascular graft [14-16].

Clinically, the presence of superficial forearm arteries can be suspected in the absence of palpable ulnar or radial pulses in their usual location, when superficial pulsatile vessels are found, or when patients complain of intermittent forearm or hand pain [1].

## Conclusion

The ulnar artery can present several anatomical variations. In this paper we describe a bilateral superficial brachioulnar artery that, instead of travelling over the anterior aspect of the forearm muscles, as is usually the case in this variant of the ulnar artery, coursed under the palmaris longus muscle, before reaching the lateral aspect of the flexor carpi ulnaris muscle and becoming part of the ulnar neurovascular bundle. This rare variant of the ulnar artery should always be born in mind when addressing the vessels of this region clinically.

## Consent

Written informed consent was obtained from the person who donated the cadaver dissected, prior to her death, for all teaching and academic purposes, namely for publication of relevant findings in scientific reports, including images. A copy of the written consent is available for review by the Editor-in-Chief of this journal.

## Competing interests

The authors declare that they have no competing interests.

## Authors’ contributions

All authors have read and approved the final manuscript. DC performed the dissection, played a major role in writing the manuscript, and analyzed the patient’s data. DP played a major role in writing the manuscript, and analyzed the patient’s data. TT aided in the editing of the manuscript, and analyzed the patient’s data. TB aided in the editing of the manuscript, and analyzed the patient’s data. LR performed the dissection, aided in the editing of the manuscript, and analyzed the patient’s data. IF performed the dissection, aided in the editing of the manuscript, and analyzed the patient’s data. SA performed the dissection, aided in the editing of the manuscript, and analyzed the patient’s data. MA played a major role in writing the manuscript, and analyzed the patient’s data. JGO played a major role in writing the manuscript, and analyzed the patient’s data.
